# Immunotherapy Frontiers in Hematology

**DOI:** 10.1097/HS9.0000000000000008

**Published:** 2017-12-20

**Authors:** Simon Hallam

**Affiliations:** St Bartholomew's Hospital, Barts Health NHS Trust, London, United Kingdom

The European Hematology Association Theme of the year 2017–2018 has rather chosen itself. Any other topic risked being swamped by the flood of excitement emanating from the field of immunotherapy. This is reflected in the recent explosion of related publications in scientific journals and eye-catching articles in the mainstream media. Not one of my outpatient clinics now passes without hopeful questions about immunotherapy from my patients and their relatives. As clinicians, we share our patients’ desperation for better outcomes. There is a widespread appetite to engage with the field, provide clarity for our patients, and contribute to progress through clinical trials.

EHA is working to satisfy this appetite through a variety of focused activities in the coming year aimed at facilitating education and empowerment of our community, as well as driving forward this exciting field at what feels like a pivotal time in blood cancer therapeutics. At the core of these activities is the recently convened Scientific Working Group (SWG) on Immune Therapies for Hematologic Disorders, chaired by Professor Hermann Einsele of the University of Würzburg, Germany, a global opinion leader in this field. The SWG will help drive cooperation on studies, and will contribute to scientific exchange at the EHA meetings as well as provide input for the EHA Learning Center (https://ehaweb.org/education/learning-center/).

Immunotherapy has, of course, been enmeshed with hematology for decades. Cytokine therapies, immune suppressants, monoclonal antibodies, and adoptive cellular therapies are well-established therapeutic partners to traditional cytotoxic chemo- and radiotherapy. But it is over the last decade that the most promising advances have been made, in strategies to directly harness T cells against hematologic cancers. These include checkpoint inhibition, bispecific T-cell engagers (BiTE), and chimeric antigen receptor T cells (CAR-T) (Fig. 1).

**Figure 1:**
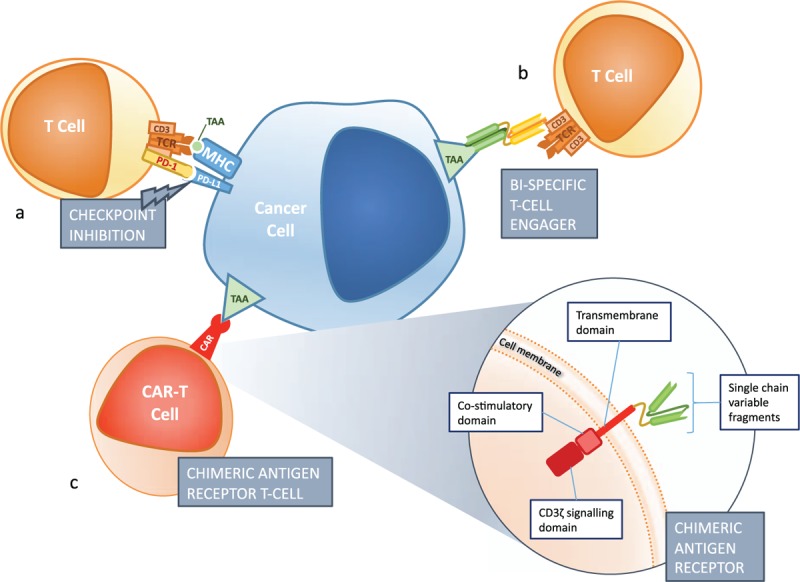
Frontiers in immunotherapy. A. Checkpoint inhibition targets the inhibitory immune checkpoints which limit T-cell activation. Antibodies targeting PD-1, PD-L1 and CTLA4 are all in clinical use. B. Bi-specific T-cell engagers, such as blinatumomab, are fusion proteins consisting of two linked single chain variable fragments. One is directed at a tumor associated antigen, and the other recruits T-cells through binding to the CD3 domain of the T-cell receptor. C. Chimeric antigen receptors consist of an extracellular, antibody derived, antigen recognition domain which targets a tumor associated antigen, a transmembrane domain, and an intracellular co-stimulatory domain linked to the signaling domain of the T cell receptor. Common co-stimulatory domains are CD28 and 4-1BB. MHC = Major histocompatability complex, PD-1 = Programmed cell death protein 1, PD-L1 = Programmed death ligand 1, TAA = Tumor associated antigen, TCR = T-cell receptor.

Checkpoint inhibition, by releasing cancer cell-suppressed T cells in waves of systemic immune activation, has shown great promise, particularly in Hodgkin lymphoma. Mixed responses in other blood cancers and excess deaths halting certain trials in myeloma have highlighted the need for further optimization of this strategy. (For a well-crafted expert tour of the field, readers would do well to study the 2016 review in *Haematologica* of Dr Santosha Vardhana and Professor Anas Younes.)^1^

BiTE, by hijacking passing T cells and forcing them to engage with tumor cells, show promise in phase 3 studies in acute lymphoblastic leukemia (ALL) with blinatumomab gaining United States Food and Drug Administration (FDA) and European Medicines Agency approval.^2^

The most excitement in recent years has been generated by CAR-T, T cells re-programmed ex vivo to recognize specific tumor-associated antigens (TAA), leading to activation and tumor killing. The remainder of this article will focus on the biology and clinical application of CAR-T technologies, directing readers to notable review articles.

## The CAR-T story

The promise of CAR-T is considerable. The most successful application has been through targeting CD19 on B-cell malignancies. This follows proof of principle with bare antibody therapies that this approach can be effective and safe. Dramatic responses in chemorefractory individuals, combined with the vast potential to refine this approach through genetic engineering of the product, have captured the imagination of cancer patients and physicians alike. CAR-T technology has also attracted vast sums of financial investment. Such is the belief that CAR-T herald a new era in cancer therapeutics, the story of their successful development is already being told. In a beautifully written commentary article in the *New England Journal of Medicine*, Dr Lisa Rosenbaum reminds us that the vision, ambition, and stubborn persistence of scientists, investors, patients, and their relatives, can drive successful translational research.^3^ Referencing a connectedness between parties with shared goals, this is a genuinely inspirational story with a much broader message than simply documenting the rise of CAR-T.

A balanced review of this field must also recognize the relative immaturity and limitations of the clinical data, and the significant adverse effects related to CAR-T infusions. Dr Jennifer Brudno and Dr James Kochenderfer have composed a state-of-the-art appraisal of this technology in *Nature Reviews Clinical Oncology*.^4^ Dr Kochenderfer writes with particular authority as one of the first to publish successful responses of B-cell malignancies to CAR-T infusion.^5^ In this review, the fundamentals of CAR-T technology are outlined, with a subsequent focus on their application to treating patients with B-cell malignancies.

The CAR comprises a fusion of an extracellular antigen recognition domain with transmembrane and intracellular T-cell signaling domains. The genetic code for the CAR construct is transferred into harvested T cells using a vector. This vector is usually a replication-incompetent retrovirus, but lentiviral or transposon systems have also been used. The antigen recognition domain is usually a single-chain variable fragment derived from a monoclonal antibody targeting a TAA.

Developments in the composition of the T-cell signaling construct impact CAR-T function and efficacy. First-generation CAR-T products use an intracellular portion of the T-cell receptor alone, with second- and third-generation products incorporating co-stimulatory domains and T-cell activation domains leading to improvements in tumor killing and CAR-T persistence.

The majority of CAR-T technologies under development use patient-derived autologous T cells harvested by leucapheresis. The optimal cell product in terms of cell dose and T-cell subset composition (unselected versus chosen ratios of CD4 and CD8 cells) remains to be elucidated. The production of autologous CAR-T products poses logistical challenges. Leucapheresis, transport to a CAR-T production unit then back to a clinical department is time-consuming, expensive, and demanding of fail-safe governance systems for this individualized cellular product. The typical current turnaround time from harvest to re-infusion is about 3 weeks, during which time the patient may progress. Universal donor or matched “off-the-shelf” allogeneic CAR-T products offer the potential advantages of reduced costs and swifter delivery from the time of request. Allogeneic CAR-T products are being used in early phase clinical trials.

Lympho-depleting conditioning regimens given before re-infusion of CAR-T, such as fludarabine with cyclophosphamide, appear necessary for optimal CAR-T survival and function. Further optimization of conditioning may improve CAR-T persistence and durability of antitumor responses. Moreover, interesting questions are being explored regarding postinfusional modification of CAR-T responses with other immunotherapies. The prospect of combining CAR-T with checkpoint inhibition might be attractive from a tumoricidal perspective, but may prove “too much of a good thing” as regards systemic immune activation.

Although autologous CAR-T products avoid the risks of graft-versus-host disease (GvHD), they are not without considerable toxicity. Infusion can precipitate a florid systemic inflammatory response, termed the cytokine release syndrome, and a range of neurologic toxicities termed CAR-T-cell-related encephalopathy syndrome. Deaths have resulted from these toxicities. Anti-interleukin 6 therapies and steroids can be effective antidotes. This may be at the cost of CAR-T persistence and efficacy. The incorporation of suicide genes encoding druggable “off-switches” also holds promise. Optimal surveillance and management of CAR-T toxicities is highly challenging and currently limits safe administration of these products to centers experienced in stem cell transplant. Dr Sattva Neelapu and colleagues address these issues in a recent expert review.^6^

Once acute toxicities have passed, disease relapse is the greatest risk. As such, the durability of responses is of great interest. Relapse is observed not only when CAR-T are no longer detectable in blood samples, but also when they persist. Antigen escape, with the survival and clinical relapse through therapeutic selection pressure of target antigen-negative clones is well described. Administering CAR-T against multiple TAA, or constructing bi-specific CAR, may mitigate this risk.

Refinement of the technology and its clinical application seem very likely to herald a new age of immunotherapy in hematology, with more patients living longer and better. There is rapid ongoing innovation in gene transfer technology and design of CAR-T constructs. Investigation and optimization of cell source and dose, conditioning, patient selection, combination with other immunotherapies, and management of toxicities, will surely herald further improvements and hopefully extension of benefit to more disease groups (Fig. 1).

## When will we see CAR-T in routine clinical practice?

In August 2017, tisagenlecleucel (Kymriah, Novartis, Basel, Switzerland), a CD19-engaging construct, became the first CAR-T product to gain FDA approval. Dr Scott Gottlieb, Commissioner of the FDA, declared this an “inflection point” in science, with advances in CAR-T therapy marking a milestone in the approach to harness immunotherapy in personalized cancer therapy.

The listed cost of Kymriah, at a headline-grabbing $475,000 for a single infusion, is itself unprecedented. This sum appears vast even when compared to the costs of competing cellular therapies such as allogeneic stem cell transplantation, even accounting for added costs of GvHD or relapse. A negotiated cost reimbursement program for a lack of response after 30 days will do little to soften the financial blow to US healthcare systems, particularly as most relapses seem to occur beyond 30 days. It must be hoped that economies of scale as regards harvest, manufacture, and distribution, allied with the impact of competition, will lead to a fall in price. Without this, CAR-T will be available outside clinical trials only to the extremely wealthy or well insured.

Kymriah has been approved for relapsed/refractory ALL in pediatric and young adult populations, in which response rates of about 80% have been observed in early phase clinical trials. Such unprecedented response rates appear to have forced changes in the approval process, with regulators seeming to deem it unethical to wait for survival data to mature. Perhaps, this poses a dilemma for our clinical community? Innovative rapid approval processes for promising new therapies in areas of urgent unmet need are very welcome. However, surely we are beholden to our patients to ensure that rigorous post-marketing studies are completed to confirm meaningful benefit and refine practice. We must recognize that current clinical outcome data from early phase studies is affected by selection bias, with corresponding “real-world” denominator numbers not yet known. High initial response rates in relapsed/refractory patients are truly exciting. We must also remain mindful that for our patients, a durable response with long-term disease-free survival is largely what constitutes true benefit. Long-term follow-up of appropriately randomized phase 3 studies is needed to establish durability of responses and long-term survival benefit.

Through promoting balanced and evidence-based discussions on CAR-T and other immunotherapy for hematologic disorders, we hope EHA can contribute not only to scientific and clinical progress in this highly promising field, but also to promote realistic and ethically sound dialogue between clinicians and patients. This promises to be a truly intriguing year.
